# Caffeine-clarithromycin coadministration and hyperlactatemia in a young infant: a case report

**DOI:** 10.62675/2965-2774.20250159

**Published:** 2025-02-27

**Authors:** Leila Costa Volpon, Flavia Maria Costa, Ana Paula de Carvalho Panzeri Carlotti

**Affiliations:** 1 Hospital das Clínicas Faculdade de Medicina de Ribeirão Preto Universidade de São Paulo São Paulo SP Brazil Division of Critical Care Medicine, Department of Pediatrics, Hospital das Clínicas, Faculdade de Medicina de Ribeirão Preto, Universidade de São Paulo - São Paulo (SP), Brazil.

**Keywords:** Apnea, Caffeine, Clarithromycin, Hyperlactatemia, Prematurity, Infant, newborn, Infant

## Abstract

Apnea is a major complication of acute respiratory tract infection in young infants and may lead to the need for ventilatory support. Caffeine is methylxanthine, which is considered the mainstay of pharmacologic treatment for apnea of prematurity. On the basis of neonatal guidelines, caffeine has been used as a respiratory stimulant for the treatment of acute respiratory tract infection-related apnea, despite low evidence of its ability to improve clinical outcomes. Hyperlactatemia has been reported in adults with caffeine poisoning. Clarithromycin acts as an inhibitor of human cytochrome P450 and may impair drug metabolism. However, there are no published data concerning lactic acidosis associated with caffeine-clarithromycin coadministration. We report a case of hyperlactatemia in a young infant born prematurely who presented to the emergency department with acute respiratory tract infection-associated apnea and who required noninvasive ventilatory support. Because respiratory viruses were not detected in the nasopharyngeal aspirates and the chest radiography revealed interstitial opacities, clarithromycin (15mg/kg/day) was started via a nasoduodenal tube. In polysomnography, dysmaturity and immaturity of the central nervous system were evident. Hence, caffeine treatment was initiated at a loading dose of 10mg/kg followed by a maintenance dose of 5mg/kg/day. After treatment initiation, the child experienced ventilatory improvement and apnea control. However, a progressive increase in the serum lactate concentration and high anion gap metabolic acidosis were observed, despite hemodynamic stability. Following discontinuation of both drugs, the serum concentrations of lactate gradually returned to normal values. Thus, clarithromycin-caffeine coadministration may cause a sharp increase in lactate concentrations and should be avoided in young infants with acute respiratory tract infection-associated apnea.

## INTRODUCTION

Apnea is a serious complication of acute respiratory tract infection (ARTI) in young infants and may lead to the need for ventilatory support. Caffeine is a methylxanthine that has been considered a pillar of the pharmacologic treatment of apnea of prematurity in recent decades.^(1)^It is metabolized primarily to paraxanthine (80%), theobromine (11%), and theophylline (5%), mainly through the cytochrome P4501A2 isozyme, and has a nonselective adenosine receptor antagonistic effect, causing increased catecholamine release by acting at presynaptic adenosine A1 receptors on the adrenal medulla.^(2)^

There are no clinical guidelines for the management of ARTI-related apnea. On the basis of neonatal guidelines, caffeine has been used as a respiratory stimulant for the treatment of ARTI-related apnea, despite low evidence of a benefit in terms of clinical outcomes.^(3)^At high doses, caffeine induces excessive sympathetic stimulation. By promoting increased glycogenolysis and lipolysis and inhibiting pyruvate dehydrogenase, caffeine increases the pyruvate concentration, which generates lactate and may cause hyperlactatemia.^(4)^

Hyperlactatemia has been reported in adults with caffeine poisoning.^(4,5)^Additionally, lactic acidosis is a known side effect of the concomitant use of clarithromycin and a calcium channel blocker.^(6)^However, there are no published data on lactic acidosis associated with caffeine-clarithromycin coadministration. We report a case of hyperlactatemia associated with caffeine-clarithromycin coadministration in a young infant with ARTI and multiple apnea episodes.

## CASE REPORT

**Figure 1:**
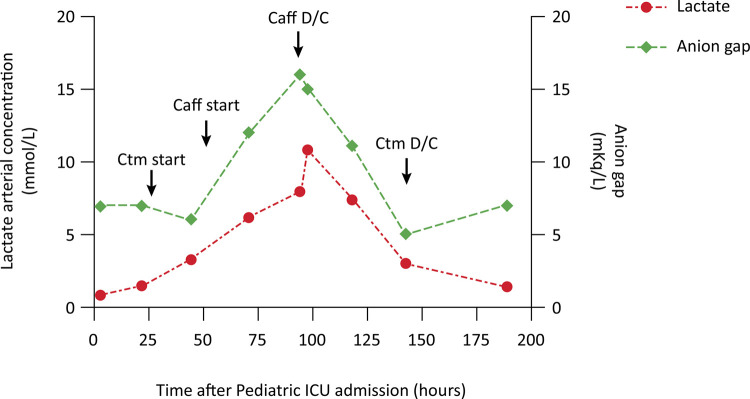
Plasma arterial lactate concentration (mmol/L) and anion gap (mEq/L) during the pediatric intensive care unit stay. Ctm - clarithromycin; Caff - caffeine; D/C - discontinuation.

An infant female of 2 months and 23 days chronological age, with a history of prematurity and a corrected age of 38 weeks and 6 days, was admitted to the emergency department with a history of food refusal, nasal obstruction, dyspnea, and multiple apnea episodes, which ultimately led to the need for hospitalization in the pediatric intensive care unit (ICU) for noninvasive ventilatory support. Because respiratory viruses were not detected in the nasopharyngeal aspirates and the chest radiography revealed interstitial opacities, clarithromycin (15mg/kg/day) was started via a nasoduodenal tube. One day later, the child experienced numerous episodes of apnea followed by oxygen desaturation and underwent polysomnography, which revealed immaturity of the central nervous system. Hence, caffeine treatment was started at a loading dose of 10mg/kg, followed by a maintenance dose of 5mg/kg/day. After treatment initiation, the child experienced ventilatory improvement and apnea control. However, a progressive increase in the arterial lactate concentration of up to 12 times the initial value and high anion gap metabolic acidosis were observed, despite hemodynamic stability. Upon pediatric ICU admission, the arterial lactate concentration was 0.9mmol/L, and it peaked at 10.8mmol/L, 48 hours after caffeine-clarithromycin coadministration (Figure 1). Other causes of increased lactate levels, such as hypoxemia and shock, were ruled out. Following discontinuation of both drugs, the serum concentrations of lactate gradually returned to normal values. Notably, the child had used caffeine for 35 days until a month before pediatric ICU admission, and the lactate concentrations over that period were all within the normal range.

## DISCUSSION

To our knowledge, this is the first report of hyperlactatemia associated with caffeine-clarithromycin coadministration. Caffeine is a very popular drug present in beverages, appetite suppressants, and various combinations of analgesic preparations. Hyperlactatemia has been reported in adult patients with caffeine poisoning due to a suicide attempt.^(2,4,5,7)^ In children, most cases reported in the literature are related to iatrogenic medication errors, accidental ingestion and child abuse.^(8,9)^Notably, none of the reported cases of caffeine poisoning in children included lactic acidosis as a possible finding.^(8,9,10)^

Apnea of prematurity is a manifestation that reflects the immaturity of the respiratory control system. In general, it resolves at approximately 36 weeks postmenstrual age, which coincides with signs of brainstem maturation. The ventilatory response to hypercapnia is significantly reduced in preterm infants and increases with increasing postnatal age. Methylxanthines are the primary treatment for apnea of prematurity because they are central stimulants, increase minute ventilation, improve CO_2_ sensitivity, decrease hypoxic depression and increase diaphragmatic activity.^(11)^

Caffeine is considered the safest methylxanthine for the treatment of apnea of prematurity because of its higher therapeutic index, better enteral absorption, and longer half-life than other methylxanthines. Caffeine is rapidly absorbed from the gastrointestinal tract after ingestion, its half-life varies between 40 hours and 230 hours in premature newborns, and the time required for the drug to reach its plasma peak is between 30 minutes and 2 hours.^(12)^ Caffeine therapy has been associated with variable side effects, including tachycardia, jitteriness, and feeding intolerance in preterm infants. Nevertheless, no clinical trial on the use of caffeine for apnea of prematurity treatment has included data regarding the serum lactate level.^(1,13)^

Clarithromycin, a bacteriostatic antimicrobial used for the treatment of upper and lower respiratory tract infections, acts as an inhibitor of human cytochrome P450, impairing the ability of drug-metabolizing enzymes. These actions can lead to adverse drug reactions due to increased levels of other concomitantly administered drugs.^(14)^There is evidence that clarithromycin can cause serious toxicity when it is administered concurrently with colchicine.^(15)^There is also a case report of ergotism associated with a drug interaction between clarithromycin and caffeine-ergotamine preparations.^(16)^In addition, coadministration of clarithromycin and verapamil may cause lactic acidosis.^(6)^In our case, the coadministration of clarithromycin and caffeine, a drug primarily metabolized by the cytochrome P450 system, may have resulted in elevated drug concentrations and hyperlactatemia. In fact, a strong positive correlation has been observed between caffeine and lactate serum concentrations in adults with caffeine poisoning.^(5)^Although we did not measure caffeine serum levels in our patient, serum lactate levels gradually decreased following the discontinuation of caffeine and clarithromycin. It is not possible to determine which of the drugs may have been primarily responsible for the increase in lactate concentrations, since both drugs were suspended almost simultaneously, but it is most likely that the interaction between the two drugs determined this side effect. Furthermore, there is a possibility that an inborn error of metabolism, not yet diagnosed, may have contributed to the patient’s clinical-laboratory picture.

## CONCLUSION

Drug‒drug interactions are important issues in clinical practice. Clinicians should be aware of the pharmacologic interactions between caffeine and drugs that act as inhibitors of the cytochrome P450 system, such as clarithromycin. Therefore, in young infants with apnea associated with acute respiratory tract infection, the coadministration of caffeine and clarithromycin should be avoided.
